# Periventricular white matter hyperintensities are associated with gait and balance in patients with minor stroke

**DOI:** 10.3389/fneur.2022.941668

**Published:** 2022-07-22

**Authors:** Chen Su, Xiaoyu Yang, Shuqi Wei, Renliang Zhao

**Affiliations:** Department of Neurology, The Affiliated Hospital of Qingdao University, Qingdao, China

**Keywords:** cerebral small vessel disease, minor stroke, total cerebral small vessel disease burden, gait speed, balance, timed-up-and-go

## Abstract

**Objective:**

Cerebral small vessel disease (CSVD) is associated with gait and balance deficits in older adults. However, the effect of CSVD-related brain injury on post-stroke mobility is unknown. This study aimed to investigate the association of CSVD with gait and balance impairment after a minor stroke.

**Methods:**

A total of 273 patients with a minor stroke (NIHSS ≤ 5 points) who were hospitalized at the Affiliated Hospital of Qingdao University were enrolled. The manifestations of white matter hyperintensities (WMH), lacunes, enlarged perivascular spaces (EPVS), and cerebral microbleeds (CMB) were statistically analyzed according to magnetic resonance imaging results, and the total burden score of CSVD was calculated. Gait function was assessed by a 6-m walking speed test, and balance function was assessed by the timed-up-and-go (TUG) test. Linear regression analysis was applied to determine the association after adjusting for key variables.

**Results:**

The correlation results showed that in patients with minor stroke, age, sex, smoking history, and the infarct site were associated with gait speed, and age and the infarct site were associated with the TUG test. In the univariate linear regression model, periventricular white matter hyperintensities (PVWMH), deep white matter hyperintensities (DWMH), and the total burden of CSVD were correlated with gait speed, while only PVWMH correlated with the TUG test. After adjusting for confounders, only PVWMH were independent predictors of gait speed (β = −0.089, *p* < 0.05) and the TUG test (β = 0.517, *p* < 0.05).

**Conclusions:**

Our study confirmed that CSVD is associated with gait and balance disorders after a minor stroke. PVWMH are independent predictors of gait and balance disorders in patients with minor stroke. These findings should be confirmed in larger prospective studies.

## Introduction

Cerebral small vessel disease (CSVD) is a pathological process in cerebral arterioles, capillaries, and venules caused by a variety of factors. It manifests in magnetic resonance imaging (MRI) as white matter hyperintensities (WMH), microbleeds (CMB), lacunes, and enlarged perivascular spaces (EPVS) (1, 2). CSVD is common in elderly individuals, and gait and balance disorders are the second most common problem in CSVD after cognitive impairment (3, 4). Gait and balance disorders can lead to falls and functional dependence, increasing the risk of hospitalization and death (5, 6). Previous studies have shown that gait and balance disorders are associated with individual MRI features of CSVD, such as WMH and CMB (7–9). Painter et al. (10) first explored and showed a correlation between the total burden of CSVD and gait impairment in 2017.

Minor ischemic stroke is an ischemic stroke in which the symptoms are only mild neurological deficits (11). A prospective cohort study showed that minor ischemic stroke is the most common form of ischemic cerebral artery disease, accounting for up to 35% of cases, and it has a 3-month recurrence rate of 19% (12). A study showed that 25–50% of patients with minor ischemic stroke have varying degrees of disability at 90 days of onset (13). The functional impairment caused by minor stroke is often ignored due to its mild symptoms, short duration, and limited rehabilitation and follow-up treatment. Therefore, its prognosis should be considered. Previous studies have shown that CSVD is associated with worse functional outcomes after stroke (14–16). The association between CSVD and gait and balance function impairment after stroke is unknown. Only one study reported that the total burden of CSVD was not associated with gait and balance after minor stroke (17).

In this study, we obtained objective assessments of gait and body balance using the 6-m walking speed test and the timed-up-and-go (TUG) test to investigate whether individual imaging markers of CSVD and the total burden can independently be used to predict gait and balance impairment in patients with minor stroke.

## Materials and methods

### Research subjects

The inpatient data of patients with acute stroke in the Affiliated Hospital of Qingdao University were collected. Inclusion criteria were as follows: patients with cerebral infarction treated within 7 days of onset (NIHSS ≤ 5); age range from 18 to 80; able to walk at least 10 m independently; complete imaging and clinical data; and informed consent of the patient. Exclusion criteria: clinical diagnosis of movement disorders such as Parkinson's disease; clear history of cerebral hemorrhage or subarachnoid hemorrhage; mental abnormality (including diagnosed depression, anxiety, or mental illness; long-term use of anti-anxiety, anti-depression, and other psychotropic drugs); other causes of WMH (such as multiple sclerosis, toxic encephalopathy, and infections); whether MRI contraindications existed; generally poor condition, complicating serious heart, liver, and lung dysfunction; associated with intracranial tumors, craniocerebral trauma; and other serious central nervous system diseases. NIHSS was performed on eligible patients after admission. Demographic data (age, sex) and vascular risk factors (hypertension, diabetes, hyperlipidemia, smoking history, and drinking history) were collected.

### Standard protocol approval, registration, and patient consent

We obtained written consent from each patient, and the Ethics Review Committee of the Affiliated Hospital of Qingdao University approved the study protocol.

### Assessment of gait and balance disorders

Gait speed was assessed by the 6-m walk speed test, a measure of motor function commonly used in clinical trials. Participants were asked to walk at a comfortable walking pace. They were asked to start walking 2 m from the start sign and stop 2 m behind the end sign to measure steady-state walking speed. Trunk balance was measured by the TUG test, a reliable, valid, and easily administered clinical tool that correlates well with the Berg Balance Scale (18) and mostly assesses functional activity after stroke (19). The test recorded the time it takes for the subject to rise to complete a 3-m walk, return to the armchair, and then resit; the longer the time, the worse the balance (20).

### Brain MRI assessment

A single HD 3.0T strong superconducting MRI scanner (GE, USA) was used to perform an MRI of the head, including T1-weighted imaging (T1WI), T2-weighted imaging (T2WI), fluid-attenuated flip recovery sequence (FLAIR), diffusion-weighted imaging (DWI), and magnetic susceptibility-weighted imaging (SWI). The MRI images were read and evaluated by two neurologists. Each read the images independently, and the final report was based on a consensus between the two. The radiologist made the final judgment in case of inconsistent assessment.

### CSVD evaluation

In this study, WMH, EPVS, CMB, and lacunes were scored using the Standards for Reporting Vascular Changes on Neuroimaging (STRIVE) (21). Lacunes were defined as ovoid lesions with a high signal around a middle-low signal on FLAIR images, 3–15 mm in diameter. WMH were high signal lesions on FLAIR and T2-weighted images, divided into two parts, periventricular white matter hyperintensities (PVWMH) and deep white matter hyperintensities (DWMH), evaluated using the Fazekas scale (0–3 points) (22). EPVS were lesions located in the basal ganglia and semioval center with the same signal intensity of cerebrospinal fluid on MRI and <3 mm in diameter. A score of 0 was assigned if EPVS = 0, 1 if EPVS ≤ 10, 2 if EPVS = 11–20, 3 if EPVS = 21–40, and 4 if EPVS ≥ 40 (23). CMB were defined as homogeneous round lesions (<10 mm) with low signals on SWI images. Total CSVD burden (0–4 points): 1 point for ≥1 lacunes; 1 point for DWMH score of 2 or 3 or PVWMH score of 3; 1 point for moderate to severe EPVS (2–4 points); and 1 point for CMB ≥1 (24).

### Statistical analysis

We used R studio (3.6.1) for statistical processing. Pearson correlation test and Spearman correlation test were used to evaluate the correlation between demographic data, vascular risk factors (such as gender, age, smoking, etc.,), NIHSS score, infarct location and CSVD, and walking speed and balance. Variables in the CSVD (including individual markers and total burden) that were significantly associated with gait and balance were subjected to linear regression analysis. Variables (except CSVD) that were statistically significant in the above correlation analysis were extracted as variables to be controlled for multiple hierarchy regression analysis. Multiple hierarchy linear regression was used to assess the independent prediction ability of individual radiographic markers and the total burden of CSVD. The statistical significance level was set to 0.05.

## Results

### Patient baseline characteristics

A total of 273 patients were enrolled during the study, including 170 men (62%) and 103 women (38%); age averaged 63.3 ± 9.2 years; gait speed averaged 1.02 ± 0.21 m/s; and TUG averaged 13.44 ± 3.44 s. Details are shown in Table 1.

**Table 1:** Baseline characters of subjects included in the study.

	***N***	**%**
Demographics	*N =* 273	
Age (years; mean ± SD)	63.3 ± 9.2	
Male	170	62.3
NIHSS (range)	0–5	
Hypertension	160	58.6
Diabetes mellitus	88	32.2
Hyperlipidemia	39	14.3
Smoking	90	33.0
Drinking	95	34.7
Infarct region
Frontal lobe	42	15.4
Parietal lobe	53	19.4
Temporal lobe	31	11.4
Occipital lobe	43	15.8
Basal ganglia	65	23.8
Thalamus	36	13.2
Corona radiate	52	19.0
Brainstem	76	27.8
Cerebellum	26	9.5
MR imaging
Lacunes (≥1)	146	53.5
CMB (≥1)	124	45.4
EPVS (≥2)	41	15.0
PVWMH Fazekas
0	26	9.5
1	104	38.1
2	108	39.6
3	35	12.8
DWMH Fazekas
0	78	28.6
1	91	33.3
2	61	22.3
3	43	15.8
Total CSVD burden scale
0	33	12.1
1	101	37.0
2	104	38.1
3	23	8.4
4	12	4.4
Speed (m/s; mean ± SD)	1.02 ± 0.21	
TUG (s; mean ± SD)	13.44 ± 3.44	

*SD, standard deviation; MR, Magnetic resonance; CMB, cerebral microbleeds; EPVS, enlarged perivascular spaces; PVWMH, periventricular white matter hyperintensities; DWMH, deep white matter hyperintensities; CSVD, cerebral small vessel disease; TUG, timed-up-and-go*.

### Univariate correlation analysis of clinical data and CSVD scores with gait and balance tests

In terms of demographic and vascular risk factors, correlation analysis showed that gait speed was significantly slower in elderly, female, and smoking patients, but only older age was associated with longer TUG duration. Regarding the NIHSS score and the infarct site, correlation analysis showed that stroke severity (NIHSS) was not related to gait speed or length of TUG test time. Infarcts at the basal ganglia and radial coronary sites were associated with slower gait speed and longer TUG test duration.

Univariate correlation analysis of CSVD imaging markers with gait and balance disturbances showed that patients with higher PVWMH scores, DWMH scores, and total CSVD burden had slower gait speed. Subjects with higher PVWMH scores spent more time on the TUG test (Table 2).

**Table 2:** Correlation analysis of gait and balance function with clinical data.

	**Gait speed**		**TUG**	
	***r***	***p***	***r***	***p***
Age	−0.263	0.000*	0.320	0.000*
Sex	0.201	0.001*	−0.108	0.074
Smoke	−0.181	0.003*	0.048	0.426
Drink	−0.112	0.065	0.032	0.600
Hypertension	−0.032	0.601	0.016	0.791
Diabetes	−0.036	0.557	−0.041	0.497
Hyperlipidemia	0.056	0.354	0.006	0.919
NIHSS	−0.016	0.792	−0.047	0.435
Infarction site
Frontal lobe	−0.011	0.861	0.066	0.278
Parietal lobe	0.037	0.541	0.118	0.051
Temporal lobe	−0.049	0.423	0.099	0.101
Occipital lobe	−0.036	0.559	0.046	0.450
Basal ganglia	−0.203	0.001*	0.315	0.000*
Thalamus	−0.084	0.167	−0.017	0.778
Corona radiate	−0.206	0.001*	0.269	0.000*
Brainstem	−0.000	0.994	−0.058	0.341
Cerebellum	0.053	0.381	−0.037	0.547
Lacunes	0.092	0.130	−0.010	0.870
PVWMH	−0.462	0.000*	0.162	0.007*
DWMH	−0.237	0.000*	0.048	0.430
EPVS	−0.051	0.401	−0.050	0.414
CMB	0.033	0.584	−0.008	0.890
CSVD total burden	−0.135	0.026*	−0.013	0.827

PVWMH, periventricular white matter hyperintensities; DWMH, deep white matter hyperintensities; CMB, cerebral microbleeds; EPVS, enlarged perivascular spaces; CSVD, cerebral small vessel disease; TUG, timed-up-and-go. ^*^P <0.05.

### Linear association of CSVD markers and total burden with gait and balance

A univariate linear regression model was used to evaluate the individual contribution of CSVD markers to gait and balance performance. PVWMH scores (β = −0.107, *P* = 0.000), DWMH scores (β = −0.043, *P* = 0.000), and the total CSVD burden (β = −0.030, *P* = 0.027) significantly predicted gait speed. PVWMH scores (β = 0.708, *P* = 0.004) predicted trunk balance (Table 3).

**Table 3:** Linear analysis of CSVD markers and total burden associated with gait and balance.

	**Gait speed**		**TUG**	
	**β**	***p***	**β**	***p***
Lacunes	ns	ns	ns	ns
PVWMH	−0.107	0.000*	0.708	0.004*
DWMH	−0.043	0.000*	ns	ns
EPVS	ns	ns	ns	ns
CMB	ns	ns	ns	ns
CSVD total burden	−0.030	0.027*	ns	ns

PVWMH, periventricular white matter hyperintensities; DWMH, deep white matter hyperintensities; CMB, cerebral microbleeds; EPVS, enlarged perivascular spaces; CSVD, cerebral small vessel disease; TUG, timed-up-and-go. ^*^P <0.05.

### Independent prediction ability of CSVD for gait and balance function

To determine the independent predictive power of CSVD for gait and balance after including demographics, vascular risk factors, infarct location, and NIHSS, a multiple hierarchical linear regression model was used for statistical analysis. Only the variables associated with gait and balance in the above statistical analysis were included in the model. For CSVD and gait speed, we included PWMH, DWMH, and CSVD total burden as coexisting predictors in the model. Model 1 only adjusted for demographic factors (age, sex), and the results showed that only PVWMH could independently predict gait speed (β = −0.090, *P* < 0.001). We added vascular risk factors (smoking) to Model 2 and showed that PVWMH independently still predicted gait speed (β = −0.086, *P* < 0.001). In Model 3, we included infarct sites based on Model 2, and the association remained significant (β = −0.089, *P* < 0.001) (Table 4) Regarding CSVD and body balance, we included PVWMH score and age in Model 1, and the results showed that PVWMH significantly independently predicted gait speed (β = 0.492, *P* = 0.041). In Model 2, we added infarct sites and found that PVWMH were still independent predictors of TUG (β = 0.517, *P* = 0.022) (Table 5).

**Table 4:** Independent predictive analysis of walking speed by CSVD in patients with minor stroke.

	**Model 1**	**Model 2**	**Model 3**
	**β(SE)**	**β(SE)**	**β(SE)**
Lacunes	ns	ns	ns
PVWMH	−0.090(0.015)*	−0.086(0.015)*	−0.089(0.015)*
DWMH	−0.020(0.012)	−0.020(0.012)	−0.020(0.011)
EPVS	ns	ns	ns
CMB	ns	ns	ns
CSVD total burden	0.008(0.013)	0.005(0.013)	0.008(0.012)
Age	−0.004(0.001)*	−0.004(0.001)*	−0.003(0.001)*
Sex	0.030(0.024)	0.050(0.024)*	0.043(0.024)
Smoke	/	−0.086(0.024)*	−0.079(0.024)*
Basal ganglia	/	/	−0.054(0.026)*
Corona radiate	/	/	−0.085(0.028) *

Model 1 adjusted for age and sex; Model 2 adjusted for age, sex and smoke; Model 3 adjusted for age, sex, smoke and infarction region. SE, standard error; PVWMH, periventricular white matter hyperintensities; DWMH, deep white matter hyperintensities; EPVS, enlarged perivascular spaces; CMB, cerebral microbleeds; CSVD, cerebral small vessel disease. *P <0.05.

**Table 5:** Independent predictive analysis of timed-up-and-go test by CSVD in patients with minor stroke.

	**Model 1**	**Model 2**
	**β(SE)**	**β(SE)**
Lacunes	ns	ns
PVWMH	0.492(0.240)*	0.517(0.224)*
DWMH	ns	ns
EPVS	ns	ns
CMB	ns	ns
CSVD total burden	ns	ns
Age	0.112(0.022)*	0.083(0.021)*
Basal ganglia	/	1.984(0.441)*
Corona radiate	/	1.967(0.475)*

Model 1 adjusted for age; Model 2 adjusted for age and infarction region. SE, standard error; PVWMH, periventricular white matter hyperintensities; DWMH, deep white matter hyperintensities; EPVS, enlarged perivascular spaces; CMB, cerebral microbleeds; CSVD, cerebral small vessel disease. *P <0.05.

## Discussion

Previous studies have shown that age-related white matter lesions in the periventricular and deep frontal lobes are associated with falls, and that deep frontal white matter lesions are associated with balance deficits in a cohort of older adults with a history of falls (7). In patients with subcortical vascular cognitive impairment, PVWMH were associated with gait scores (9). Regional brain white matter injury is associated with gait and balance deficits in patients with Alzheimer's disease (25). A follow-up of up to 13 years in a longitudinal study showed that increased and progressive total WMH and PVWMH load were associated with decreased gait function in cognitively normal adults (26). In community-dwelling older adults, WMH and the total CSVD burden are major drivers of gait impairment but are not associated with balance impairment (10). In older patients with CSVD, CMB in temporal, frontal, and basal ganglia regions was associated independently of other CSVD markers with gait and balance impairment (8). In community-dwelling patients without neurological diseases, total CSVD burden, WMH, CMB, and lacunes were independently associated with gait and balance impairment (27). Recently, a prospective study included 200 patients with minor stroke (NIHSS ≤ 7 points) and divided them into lacunar and non-lacunar stroke groups based on MRI findings. The researchers objectively measured patients' gait balance impairment by the TUG test 3 years after stroke, subjectively assessed by the Stroke Impact Scale activity domain score, and tested the relationship between the total CSVD burden scale and functional stroke outcomes in a secondary analysis. The results showed that in the overall minor stroke population, total CSVD burden was not associated with TUG, Stroke Impact Scale scores, or Modified Rankin Scale scores 3 years after stroke; in patients with non-lacunar stroke, total CSVD burden was only associated with subjective activity impairment (17). However, the study sample size was small, and selection bias during clinical follow-up may be large.

Selection bias in cohort studies refers to the bias in the study that results from improper selection of study subjects. Loss to follow-up bias is a bias caused by study subjects dropping out of the cohort for various reasons. Loss to follow-up bias is also essentially selection bias (28). The study had a long follow-up period, so it is possible that patients with significant disabilities did not participate in the clinical follow-up. The study had a missed follow-up rate of >20%, so the loss to follow-up bias was large (17). To control for missing visit bias, researchers should inquire whether the missing person has died and the cause of death, and compare information on certain characteristics obtained at the baseline survey between the missing and non-missing subjects. However, the best way to control for loss to follow-up bias is to reduce missing subjects as much as possible and to improve the compliance of study subjects (29). This is a cross-sectional study, and there may be a non-response bias in selection bias. Non-response bias refers to bias due to the unwillingness of survey respondents to cooperate or their inability to participate in the survey for other reasons (28). The control for this bias should focus on improving the compliance and acceptance rate of the study subjects (30).

Minor stroke is common, and a growing number of epidemiological studies suggest that patients with minor stroke have some degree of physical and cognitive dysfunction, leading to a reduced quality of life (31, 32). Our study found that the presence of PVWMH is the most important predictor of gait and balance disorders in patients with minor stroke. PVWMH, DWMH, and the total CSVD burden were correlated with gait speed in the regression model, but only PVWMH explained the changes in TUG. After adjusting for the effects of demographics, vascular risk factors, NIHSS, and infarct sites on gait and balance, PVWMH significantly predicted gait speed and TUG time, independent of other radiographic markers and overall CSVD burden. The severity of gait and balance disorders was associated with infarct sites but not with stroke severity (NIHSS). After excluding the effect of cerebral infarction in this study, PVWMH remained an independent predictor of gait and balance and was independent of other CSVD markers.

The present study demonstrated the effect of PVWMH on gait and balance deficits in subjects, which is in general agreement with previous studies (9, 26). Unlike previous studies, the present study did not show the independent predictive power of lacunes (27) and CMB (8) on gait deficits. Although the total CSVD burden may better reflect the overall effect of CSVD on the whole brain, (33, 34) the present study did not show an independent effect of total CSVD burden on gait and balance. Possible reasons for the dissimilar results are as follows. First, the population included in our study were patients with acute ischemic minor stroke, and not the community population included in most studies. We had to take into account the limitation of mobility caused by the infarctions, which made CSVD less sensitive to the effects of gait and balance. Second, the subjects included in our study were relatively young, with an average age of 63.3 years. Third, we assessed the burden of CSVD with a visual rating scale and mobility impairment with a 6-m walking test and a TUG test. In addition, the present study showed that in the acute phase of cerebral infarction, only infarcts in the basal ganglia and radial crown sites were associated with gait and balance, while failing to confirm the idea that sites such as the cerebellum and frontoparietal lobe are involved in motor and ataxic regulation (35). This may be related to the small sample size included in the study.

This is the first study to investigate the effects of individual imaging markers of CSVD on gait and balance disorders in patients with minor stroke, and only one study mentioned above reported an association between overall CSVD burden and gait (17). PVWMH had significant independent predictive power after considering the effects of age, sex, vascular risk factors, stroke severity, and infarct location on gait and balance disorders. Gait is a highly cognitive process, and the poor gait performance caused by CSVD is partly mediated by cognitive function (36). PVWMH are more likely to cause cognitive damage because the periventricular white matter contains a large concentration of neurons and fibers related to learning, memory, and cognition. The white matter bundles passing through the periventricular area are denser (37). PVWMH can also directly damage motor pathways, leading to decreased gait and trunk stability (38, 39).

Our study had some limitations. First, our sample size was relatively small for stroke, a common disease. Large-scale studies are needed to determine the association between CSVD and gait and balance disorders in patients with minor stroke. Second, although we adjusted the results for stroke, the infarct sites are bound to have an impact on the clinical manifestations caused by CSVD in the acute stage of stroke. Third, our study was cross-sectional, and a causal relationship between CSVD and gait and balance disorders cannot be confirmed.

In summary, this study is the first to examine the association of individual markers and the total burden of CSVD with gait and balance impairment in patients with minor stroke. The findings show that PVWMH are independently associated with gait speed and trunk balance in patients with minor stroke. The PVWMH score is a possible independent marker to identify minor stroke patients at risk for gait and balance disorders. These findings should be confirmed in future large studies. It is suggested that in clinical work, we should focus on prevention strategies directed against the progression of WMH, which may provide useful assistance in the prevention and treatment of gait and balance dysfunction in patients with minor stroke.

## Data availability statement

The original contributions presented in the study are included in the article/Supplementary Material, further inquiries can be directed to the corresponding author.

## Ethics statement

The studies involving human participants were reviewed and approved by the Ethics Review Committee of the Affiliated Hospital of Qingdao University. Written informed consent for participation was not required for this study in accordance with the national legislation and the institutional requirements.

## Author contributions

RZ planned the study. CS, XY, and SW analyzed the data and edited the manuscript. CS wrote the manuscript. All authors contributed to the article and approved the submitted version.

## Conflict of interest

The authors declare that the research was conducted in the absence of any commercial or financial relationships that could be construed as a potential conflict of interest.

## Publisher's note

All claims expressed in this article are solely those of the authors and do not necessarily represent those of their affiliated organizations, or those of the publisher, the editors and the reviewers. Any product that may be evaluated in this article, or claim that may be made by its manufacturer, is not guaranteed or endorsed by the publisher.
